# Internship experience and psychological symptoms among Chinese healthcare trainees: evidence from structural equation modeling

**DOI:** 10.3389/fmed.2026.1760463

**Published:** 2026-07-14

**Authors:** Xiaomin Xu, Guanguan Qiu, Oushan Tang, Guoping Zheng

**Affiliations:** Department of Medical Education, Shaoxing Second Hospital (The Second Affiliated Hospital of Shaoxing University), Shaoxing, Zhejiang, China

**Keywords:** anxiety, burnout, depression, internship experience, structural equation modeling

## Abstract

**Objective:**

This study aimed to investigate the pathways linking internship experience to psychological symptoms among Chinese healthcare trainees, with a particular focus on the mediating role of burnout.

**Methods:**

A single-center cross-sectional survey was conducted among 241 Chinese healthcare trainees at a teaching hospital. Data were collected using a self-developed Internship Experience Scale, the Maslach Burnout Inventory–Student Survey (MBI-SS), and the Symptom Checklist-90 (SCL-90). Structural equation modeling (SEM) was applied to examine the hypothesized relationships, and mediation effects were tested using bootstrap procedures.

**Results:**

Internship experience was negatively associated with burnout, whereas burnout showed positive associations with anxiety and depression. Anxiety and depression were highly correlated, and mediation analysis confirmed burnout as a significant intermediary linking internship experience and psychological symptoms.

**Conclusion:**

This study highlights the critical role of burnout in shaping the mental health outcomes of healthcare trainees, emphasizing the need for supportive educational environments to foster resilience and well-being.

## Introduction

Numerous recent studies have reported high prevalence rates of anxiety, depression, and burnout among healthcare trainees, often exceeding those observed in the general population (1). A seminal meta-analysis by Rotenstein et al. found a global prevalence of depression or depressive symptoms among medical students of 27.2%. More recent evidence suggests these rates may be even higher. A 2026 scoping review of systematic reviews reported prevalence ranges of 18.1%–50.0% for depression and 17%–54% for anxiety among medical students worldwide (2). During the COVID-19 pandemic, a meta-analysis estimated the pooled prevalence of anxiety and depression at 45% and 48%, respectively. Consistently, studies focusing on Chinese healthcare trainees have also confirmed a high psychological burden, with a national meta-analysis reporting a depression prevalence of 29% (3). These symptoms often emerge or intensify during the clinical internship phase (4, 5).

Internship marks a transition from classroom learning to direct patient care, presenting interns with a series of new stressors. Research has consistently shown that this stage is characterized by a sudden increase in clinical responsibility, which can trigger anxiety, undermine confidence, and increase the risk of junior doctors’ burnout (6). Interns often experience a “reality shock” when transitioning between the theoretical knowledge they acquired at medical school and the practical requirements of the workplace (7). This transition is further complicated by role conflicts - the tension between working as a learner and a medical provider simultaneously - which can lead to feelings of inadequacy and fatigue (8). Given the diverse composition of the clinical training population (undergraduate medical students, nursing students, etc.), the nature and intensity of the stress faced during these transitional stages may vary, so detailed research is necessary.

Beyond the structural demands of training, students’ subjective perceptions of their internship experience play a pivotal role in shaping mental health outcomes. Stressors such as inadequate supervisory support, strained doctor-patient interactions, and lack of autonomy have been linked to poor psychological adaptation (9). Existing literature, however, has predominantly emphasized isolated stress factors or singular psychological outcomes. For example, prior studies have focused on specific contributors such as workload, academic pressure, or doctor–patient interactions, often examining their independent associations with anxiety or depression rather than considering a comprehensive framework (10–12). Few studies have systematically conceptualized internship experience as an integrated construct or explored its comprehensive influence on the mental health of healthcare trainees (13, 14).

Burnout, characterized by emotional exhaustion, depersonalization, and reduced professional efficacy, may represent a key mechanism connecting internship experience to psychological symptoms (15). Empirical evidence suggests that burnout is highly prevalent in healthcare trainees and strongly associated with both anxiety and depressive symptoms. Theoretically, a negative internship experience may foster emotional depletion and cynicism, which in turn elevate the risk of psychopathological outcomes (16, 17). Nevertheless, prior investigations have largely relied on correlation or regression analyses. Studies employing structural equation modeling (SEM) to capture the complex pathways between internship experience, burnout, and mental health remain scarce.

In light of these gaps, the present study aimed to examine the pathways linking internship experience to anxiety and depressive symptoms among Chinese healthcare trainees, with burnout as a potential mediator. By introducing a novel Internship Experience Scale and applying SEM, this work provides an integrative framework to understand how educational environments shape psychological well-being. The findings are expected to inform targeted interventions that optimize internship design and support systems to promote healthier training trajectories.

## Materials and methods

### Study design and participants

**FIGURE 1:**
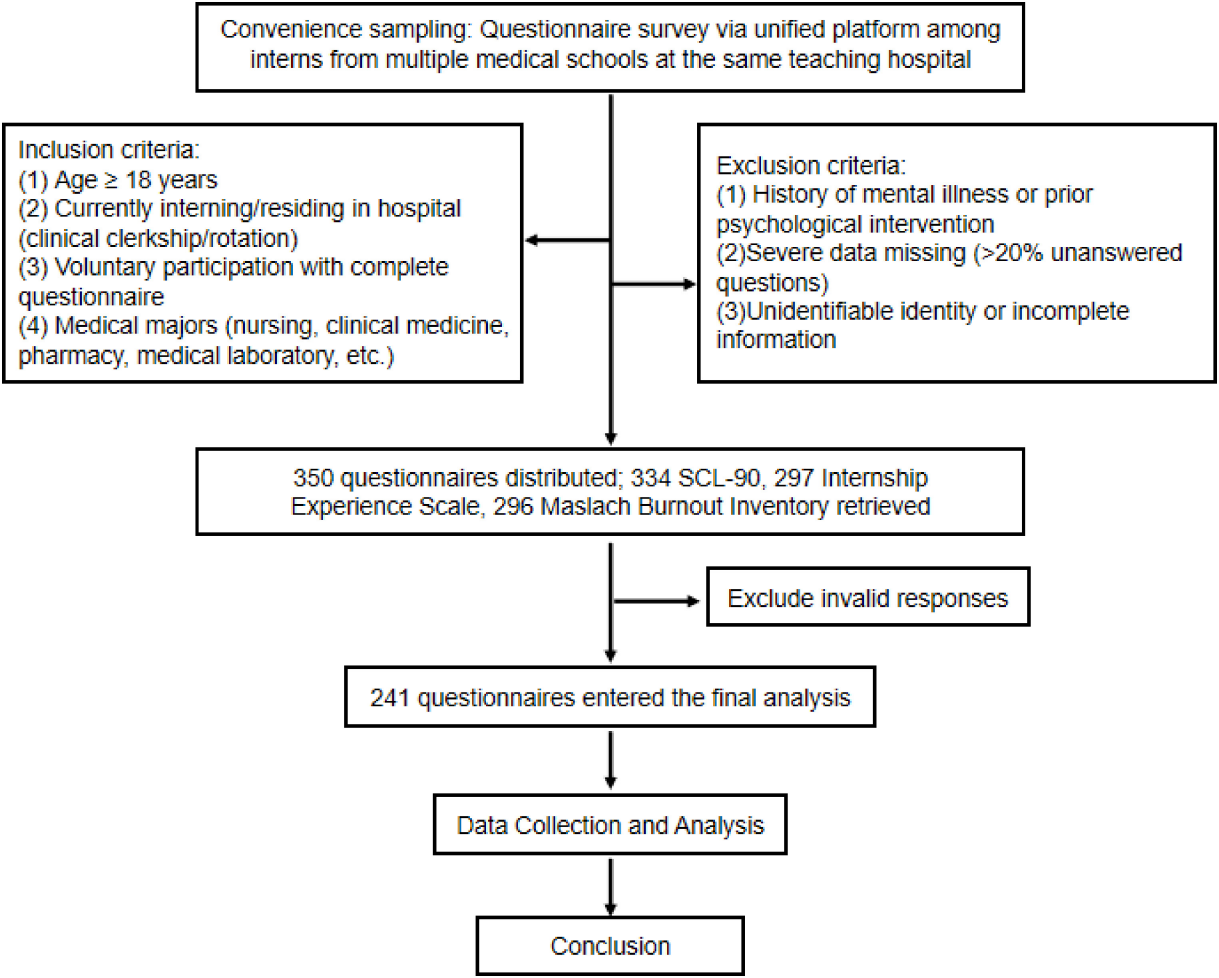
Research subject flowchart.

This study adopted a single-center cross-sectional survey design conducted at a teaching hospital in China. The participants were medical students from different secondary vocational schools, junior colleges, and undergraduate institutions in China who were undertaking clinical internships or standardized residency training at the same teaching hospital. Convenience sampling was employed, and data were collected via an online questionnaire platform between July and August 2025. Among 350 distributed questionnaires, valid responses were received from 334 for the SCL-90, 297 for the Internship Experience Scale, and 296 for the MBI-SS. After excluding invalid responses, 241 complete and eligible questionnaires were included in the final analysis (Figure 1).

Inclusion criteria: (1) age ≥16 years; (2) currently engaged in clinical internship or standardized training, at either the clerkship or rotation stage; (3) voluntary participation with completion of all required questionnaire items; and (4) major in nursing, clinical medicine, pharmacy, medical laboratory science, or other health-related disciplines. Exclusion criteria: (1) a history of diagnosed psychiatric disorders or previous psychological interventions; (2) severe missing data (>20% of unanswered items); or (3) incomplete or unidentifiable demographic information.

Sampling procedure: Due to the practical limitations in recruiting students during clinical internships, the convenience sampling method was adopted. During the study period, all medical interns who had completed internships or standardized residency training at the teaching hospital and met the eligibility criteria were invited to participate through the institutional communication channels (hospital management department, internship supervisor). Participation was voluntary and anonymous, and no incentives were provided. This method enabled a wide coverage of interns from multiple educational institutions.

### Development and validation of the Internship Experience Scale

The Internship Experience Scale was developed to capture medical trainees’ subjective perceptions of stressors and support during clinical internships in the Chinese context. The initial item pool was generated based on a comprehensive literature review of factors influencing medical student well-being, including workload, supervisory support, and emotional demands (5, 18–21). Twelve items were drafted using a five-point Likert scale (1 = strongly disagree to 5 = strongly agree), with higher scores indicating more positive experiences (i.e., better internship quality). Reverse-coded items were included to balance response sets.

Content validity was assessed by a panel of six experts (three in medical education and three in clinical psychology). Each expert rated the relevance of each item to the construct of internship experience using a four-point scale (1 = not relevant to 4 = highly relevant). The content validity index (CVI) was calculated for each item; items with CVI < 0.80 were revised or removed. This process resulted in a seven-item scale comprising two domains: task-related stress (four items, e.g., “Internship tasks leave me physically and emotionally exhausted”) and teaching support (three items, e.g., “I receive adequate guidance from my supervisors during internship”).

A pilot test was conducted with 35 medical trainees (not included in the main study) to assess clarity, comprehensibility, and feasibility. Minor wording adjustments were made based on participant feedback. The preliminary Cronbach’s α for the scale was 0.88, indicating good internal consistency.

In this study sample, the scale demonstrated good reliability and initial validity. The Cronbach’s α of the total scale was 0.907 (as shown in Table 1), indicating a high degree of internal consistency (α > 0.70).

**TABLE 1:** Reliability analysis of each scale dimension.

Scale	Alpha	Mean	SD
Internship experience	0.907	4.15	0.63
MBI_EE	0.94	2.71	1.47
MBI_DP	0.912	1.93	1.1
MBI_PA	0.934	2.02	1.14
SCL_90	0.966	10.98	3.22

### Measures

Three validated instruments were applied in this study. The Internship Experience Scale was a self-developed tool consisting of seven items that assessed two domains, namely task-related stress and teaching support. Each item was rated on a five-point Likert scale, with higher scores indicating a more favorable perception of the internship. Burnout was measured using the MBI-SS (22), which captures the dimensions of emotional exhaustion, cynicism, and professional efficacy. Responses were recorded on a seven-point Likert scale ranging from “never” to “always,” where higher values reflected greater burnout. Psychological symptoms were evaluated with the SCL-90 (23, 24), which is a well-validated multi-dimensional tool with the advantage of capturing a broader range of psychological symptoms (25). It is widely used among Chinese medical students (26). Although specific tools like PHQ-9 or GAD-7 focus on diagnostic screening, the SCL-90 is capable of assessing the severity of symptoms on a continuous scale, which is highly suitable for conducting structural equation model analysis in non-clinical populations. This instrument comprises 90 items covering nine domains of psychological distress, and in this study particular attention was given to the depression and anxiety subscales. Each item was scored on a five-point Likert scale, with higher scores corresponding to greater symptom severity.

### Reliability and validity

The reliability of each scale and its subscales was assessed using Cronbach’s α coefficients, with values above 0.70 generally considered indicative of acceptable internal consistency. To further evaluate construct validity, factor analytic methods were applied, including exploratory and confirmatory factor analyses, in order to verify the dimensional structure of the instruments.

### Statistical analysis

All statistical analyses were conducted using IBM SPSS Statistics 22.0 (IBM Corporation, Armonk, New York, USA), including descriptive statistics, between-group comparisons (independent sample *t*-tests or one-way ANOVA), and Pearson correlation analysis. The structural equation model (SEM) was analyzed using IBM SPSS Amos 24.0 (IBM Corporation, Armonk, New York, USA). Within the Amos framework, the mediating effect was tested using the bootstrap method, with 5000 samples drawn. Group analysis was performed using non-parametric tests (Kruskal-Wallis test or Wilcoxon rank sum test). Statistical significance was set at a two-tailed *p*-value < 0.05.

## Results

### Baseline characteristics of participants

A total of 241 students were included in the final analysis (Table 2). The sample was predominantly female (*n* = 169, 70.1%), with males representing 29.9% (*n* = 72). The mean age of participants was 20.77 ± 1.75 years. With respect to educational background, nearly half of the students were enrolled in bachelor’s degree programs (*n* = 117, 48.6%), followed by those from junior colleges (*n* = 93, 38.6%) and higher vocational programs (*n* = 31, 12.9%). In terms of academic specialization, nursing accounted for the largest proportion (*n* = 120, 49.8%), followed by clinical medicine (*n* = 44, 18.3%) and medical laboratory technology (*n* = 21, 8.7%), whereas each of the remaining majors accounted for less than 6% of the cohort.

**TABLE 2:** Baseline characteristics of all participants.

Variable	Item (*n* = 241)	Summary
Major	Nursing	120 (49.79%)
	Radiological medicine	1 (0.41%)
	Medical imaging technology	13 (5.39%)
	Psychiatry	12 (4.98%)
	Clinical medicine	44 (18.26%)
	Pharmacy	4 (1.66%)
	Rehabilitation therapy	5 (2.07%)
	Traditional Chinese medicine	3 (1.24%)
	Medical laboratory technology	21 (8.71%)
	General medicine	5 (2.07%)
	Midwifery	6 (2.49%)
	Stomatology	1 (0.41%)
	Medical information engineering	2 (0.83%)
	Health services and management	1 (0.41%)
	Anesthesiology	1 (0.41%)
	Biomedical engineering	2 (0.83%)
Gender	Male	72 (29.88%)
	Female	169 (70.12%)
Education	Higher vocational	31 (12.86%)
	Junior college	93 (38.59%)
	Bachelor’s degree	117 (48.55%)
MBI_EE_Total		13.56 ± 7.35
MBI_DP_Total		7.71 ± 4.39
MBI_PA_Total		12.13 ± 6.85
Internship experience_Total		29.07 ± 4.44
Age		20.77 ± 1.75
Depression_Total		16.21 ± 5.23
Anxiety_Total		12.14 ± 3.86

Regarding study variables, the mean total score for internship experience was 29.07 ± 4.44. Burnout levels, as measured by the MBI-SS, averaged 13.56 ± 7.35 for emotional exhaustion, 7.71 ± 4.39 for cynicism, and 12.13 ± 6.85 for professional efficacy. Psychological symptoms assessed by the SCL-90 indicated mean depression and anxiety scores of 16.21 ± 5.23 and 12.14 ± 3.86, respectively.

### Comparisons of internship experience and psychological symptoms across demographic groups

Internship experience differed significantly by age (*p* = 0.002). Students aged 16–18 years reported the highest scores (31.27 ± 3.27), compared with 29.13 ± 4.56 for those aged 19–21 years and 28.24 ± 4.39 for those aged 22–25 years. Psychological symptoms showed the opposite trend, with younger students exhibiting higher SCL-90 total scores (123.97 ± 47.79 vs. 108.36 ± 28.48 and 106.95 ± 29.69; *p* = 0.040). Education level was also associated with internship experience (*F* = 10.128, *p* = 0.002), with higher vocational students reporting the most favorable perceptions (30.90 ± 3.74) and bachelor’s students the least (28.27 ± 4.30). In contrast, no significant differences in either internship experience or psychological symptoms were observed between male and female students or across academic majors (Table 3).

**TABLE 3:** Comparison of SCL-90 scores and internship experience scores with demographic data.

Characteristic	Case (*N* = 241)	Internship experience_total	t/F	*P*	SCL_total score	t/F	*P*
Age (y)			9.798	0.002		4.278	0.04
16–18	30	31.27 ± 3.27			123.97 ± 47.79		
19–21	123	29.13 ± 4.56			108.36 ± 28.48		
22–25	88	28.24 ± 4.39			106.95 ± 29.69		
Gender			−0.819	0.414		0.658	0.511
Male	72	28.68 ± 5.09			111.82 ± 30.53		
Female	169	29.24 ± 4.13			108.92 ± 32.94		
Education			10.128	0.002		3.475	0.064
Higher vocational	31	30.9 ± 3.74			121.55 ± 45.42		
Junior college	93	29.46 ± 4.62			108.9 ± 32.49		
Bachelor’s degree	117	28.27 ± 4.3			107.38 ± 27.04		
Major			0.063	0.803		0.15	0.699
Nursing	120	29.57 ± 4.45			110.74 ± 35.93		
Radiological medicine	1	25 ± NA			127 ± NA		
Medical imaging technology	13	28.54 ± 3.82			111.15 ± 26.58		
Psychiatry	12	30.42 ± 3.87			112 ± 32.72		
Clinical medicine	44	26.75 ± 4.68			103.57 ± 20.91		
Pharmacy	4	33.25 ± 2.06			120.5 ± 37.75		
Rehabilitation therapy	5	31.8 ± 3.27			111.6 ± 25.44		
Traditional Chinese medicine	3	30.33 ± 2.08			93 ± 5.2		
Medical laboratory technology	21	28.62 ± 4.09			115.48 ± 26.26		
General medicine	5	27.4 ± 3.71			126.4 ± 77.52		
Midwifery	6	29.5 ± 3.21			105.33 ± 27.6		
Stomatology	1	35 ± NA			90 ± NA		
Medical information engineering	2	28 ± 0			110.5 ± 23.33		
Health services and management	1	35 ± NA			96 ± NA		
Anesthesiology	1	27 ± NA			90 ± NA		
Biomedical engineering	2	34.5 ± 0.71			96 ± 8.49		

To further evaluate the generalizability of the Internship Experience Scale, subgroup analyses were conducted across gender, age groups, and educational levels. The results showed that the scale demonstrated consistent discriminatory ability across different subgroups. Significant differences in both task-related stress and teaching support scores were observed across age groups (*p* = 0.009 and *p* = 0.012, respectively) and educational levels (*p* = 0.004 and *p* = 0.013, respectively), indicating that the scale was sensitive to variations in internship experiences among trainees with different backgrounds. In contrast, no significant differences were observed between male and female participants (*p* = 0.14 for task-related stress and *p* = 0.90 for teaching support), suggesting that the scale performs consistently across gender. These findings support the robustness and general applicability of the scale in heterogeneous trainee populations (Supplementary Tables 1–3).

### Comparisons of burnout dimensions across demographic groups

Significant age-related differences were observed in all three dimensions of burnout (Table 4). Emotional exhaustion increased progressively with age, from 10.23 ± 5.32 in students aged 16–18 years to 13.25 ± 7.33 in those aged 19–21 years and 15.11 ± 7.61 in the 22–25 year group (*p* = 0.001). Similar trends were noted for cynicism (5.93 ± 2.74, 7.39 ± 4.32, and 8.76 ± 4.70, respectively; *p* = 0.001) and for reduced professional efficacy (8.93 ± 4.83, 12.40 ± 6.99, and 12.84 ± 6.99; *p* = 0.023).

**TABLE 4:** Comparison of demographic variables and burnout scores.

Characteristic	Case (*N* = 241)	MBI_EE_Total	t/F	*P*	MBI_DP_Total	t/F	*P*	MBI_PA_Total	t/F	*P*
Age (y)			10.366	0.001		11.052	0.001		5.247	0.023
16–18	30	10.23 ± 5.32			5.93 ± 2.74			8.93 ± 4.83		
19–21	123	13.25 ± 7.33			7.39 ± 4.32			12.4 ± 6.99		
22–25	88	15.11 ± 7.61			8.76 ± 4.7			12.84 ± 6.99		
Gender			1.382	0.169		0.163	0.871		−0.728	0.468
Male	72	14.57 ± 7.5			7.78 ± 4.11			11.65 ± 6.45		
Female	169	13.12 ± 7.26			7.68 ± 4.52			12.33 ± 7.02		
Education			22.433	<0.001		12.301	0.001		7.859	0.005
Higher vocational	31	10.84 ± 5.9			6.29 ± 3.16			10 ± 6.03		
Junior college	93	11.48 ± 6.38			6.9 ± 4.12			11.34 ± 6.67		
Bachelor’s degree	117	15.92 ± 7.71			8.73 ± 4.66			13.32 ± 7.01		
Major			2.524	0.113		1.793	0.182		1.536	0.216
nursing	120	11.62 ± 6.42			6.63 ± 3.9			10.84 ± 6.23		
Radiological medicine	1	25 ± NA			10 ± NA			6 ± NA		
Medical imaging technology	13	17.08 ± 7.74			10.77 ± 5.46			16.92 ± 8.2		
Psychiatry	12	16.92 ± 9.97			7.92 ± 4.48			12.58 ± 8.53		
Clinical medicine	44	16.64 ± 7.06			9.89 ± 4.57			13.95 ± 6.79		
Pharmacy	4	9.5 ± 2.89			6 ± 2.31			11.25 ± 6.7		
Rehabilitation therapy	5	12 ± 7.94			8 ± 3.74			10.4 ± 5.13		
Traditional Chinese medicine	3	5 ± 0			4 ± 0			6 ± 0		
Medical laboratory technology	21	18.38 ± 7.57			8.95 ± 4.76			15.29 ± 6.52		
General medicine	5	11 ± 6.52			7.8 ± 5.02			13 ± 9.54		
Midwifery	6	13 ± 5.06			6.67 ± 4.18			12.33 ± 8.96		
Stomatology	1	5 ± NA			4 ± NA			6 ± NA		
Medical information engineering	2	11 ± 0			7 ± 0			9 ± 0		
Health services and management	1	5 ± NA			4 ± NA			6 ± NA		
Anesthesiology	1	13 ± NA			9 ± NA			12 ± NA		
Biomedical engineering	2	6.5 ± 2.12			4 ± 0			7 ± 1.41		

Educational attainment was also significantly associated with burnout. Bachelor’s students reported the highest scores for emotional exhaustion (15.92 ± 7.71), cynicism (8.73 ± 4.66), and reduced professional efficacy (13.32 ± 7.01), compared with junior college students (11.48 ± 6.38, 6.90 ± 4.12, and 11.34 ± 6.67, respectively) and vocational students (10.84 ± 5.90, 6.29 ± 3.16, and 10.00 ± 6.03). The differences were statistically significant for all three dimensions (EE: *p* < 0.001; DP: *p* = 0.001; PA: *p* = 0.005). In contrast, no significant differences in burnout dimensions were found between male and female students or across academic majors (all *p* > 0.05).

### Reliability and construct validity of measurement instruments

Exploratory factor analysis (EFA) was conducted to examine the underlying structure of the Internship Experience Scale. Two factors were extracted using principal axis factoring with oblique rotation, explaining 73.6% of the total variance. The first factor accounted for 49.7% of the variance, and the second factor accounted for 23.8%. All items demonstrated substantial loadings on their corresponding factors, with standardized factor loadings ranging from 0.705 to 0.954. Items Q1–Q2 loaded on the task-related stress factor, while items Q3–Q7 loaded on the teaching support factor. Cross-loadings were minimal (maximum cross-loading = 0.13), indicating a clear and well-defined factor structure (Supplementary Table 4). Confirmatory factor analysis (CFA) was further performed to validate the factor structure identified by EFA. The two-factor model demonstrated an acceptable fit to the data, with χ^2^/df = 5.83, CFI = 0.953, TLI = 0.925, SRMR = 0.042, and RMSEA = 0.142. All standardized factor loadings were statistically significant (*p* < 0.001) and ranged from 0.774 to 0.949, further supporting the construct validity of the scale. The internal consistency of the scale was high, with a Cronbach’s α coefficient of 0.907 for the total scale. Subscale reliabilities were also acceptable, 0.940 for emotional exhaustion, 0.912 for cynicism, 0.934 for professional efficacy, and 0.966 for the SCL-90, all exceeding the accepted threshold of 0.70 and indicating strong internal consistency. Mean scores suggested moderate levels of burnout and psychological symptoms in the cohort, with the Internship Experience Scale averaging 4.15 ± 0.63, burnout dimensions ranging from 1.93 ± 1.10 to 2.71 ± 1.47, and the SCL-90 showing a mean score of 10.98 ± 3.22. The relatively elevated RMSEA value may be attributable to sample size sensitivity and model complexity, whereas other fit indices (CFI, TLI, and SRMR) consistently indicated acceptable model fit, supporting the overall adequacy of the measurement model.

### Correlations among internship experience, burnout, and psychological symptoms

**FIGURE 2:**
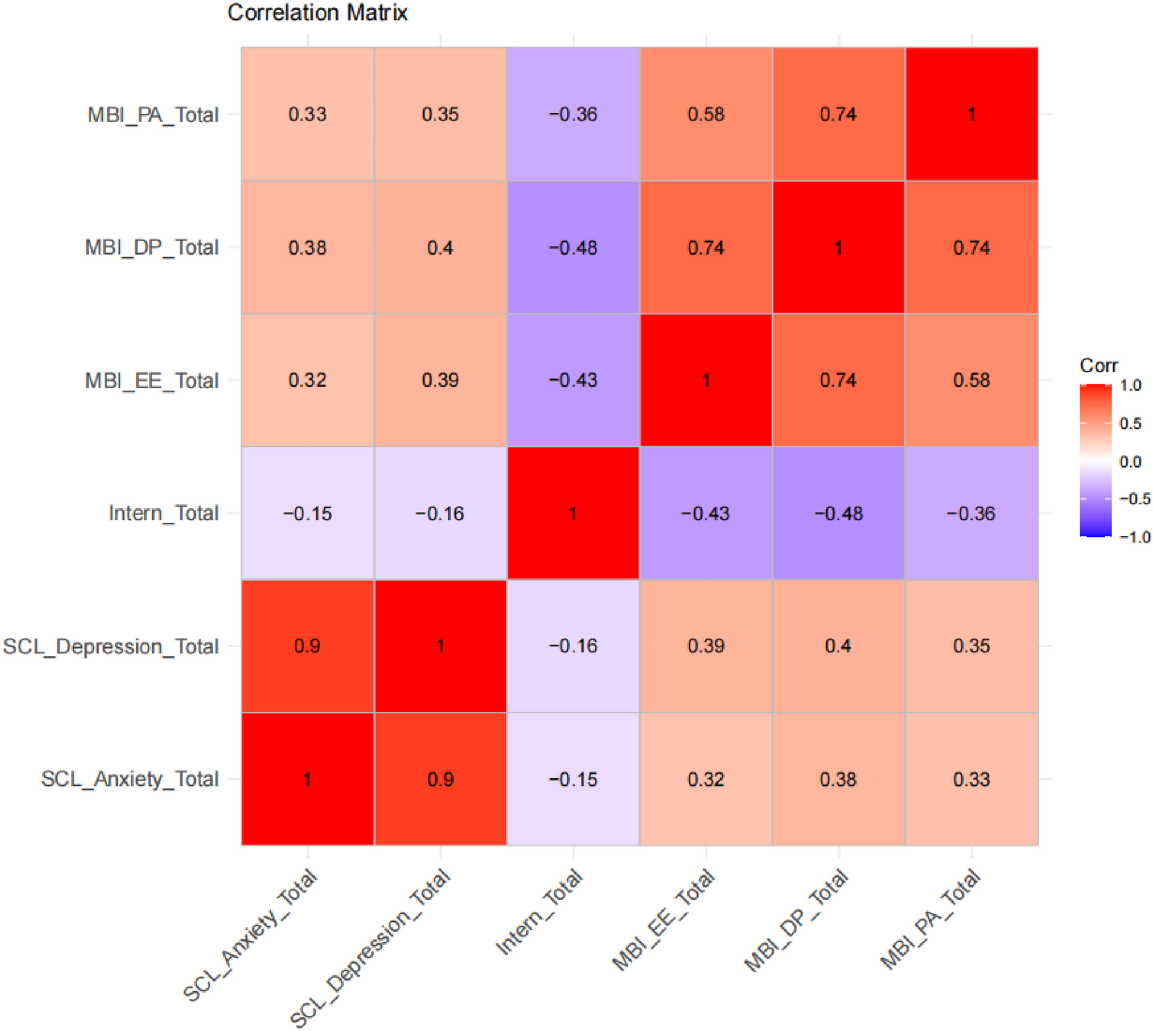
Correlation matrix among internship experience, burnout dimensions, and psychological symptoms. Pearson correlation coefficients are presented, with color intensity indicating the strength and direction of the association (red = positive, blue = negative).

As shown in Figure 2, anxiety and depression were highly correlated (*r* = 0.90), indicating a strong comorbidity between these psychological symptoms. Internship experience was negatively associated with all three dimensions of burnout, with moderate correlations observed particularly for emotional exhaustion (*r* = −0.43) and cynicism (*r* = −0.48). Furthermore, burnout dimensions showed significant positive correlations with both anxiety and depression (*r* = 0.32–0.40).

### Structural equation modeling of hypothesized pathways

**FIGURE 3:**
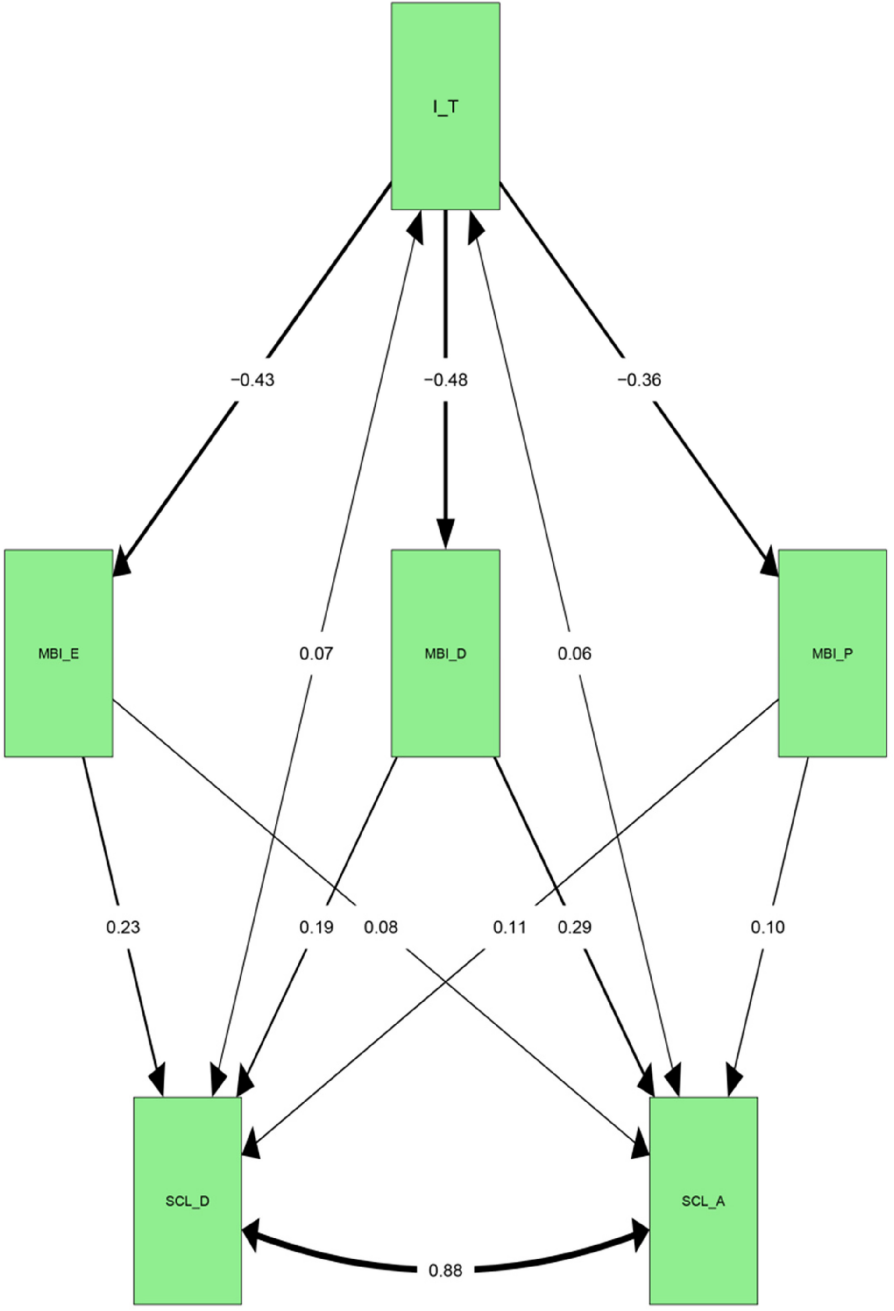
Structural equation model of internship experience, burnout dimensions, and psychological symptoms. Standardized path coefficients (β-values) are displayed along the arrows.

The structural equation model has a very good to excellent fit with the data. Its CFI value is 0.985, the RMSEA value is 0.080, and the SRMR value is 0.058. All these indicators have met the common standards for the applicability of the structural equation model. The structural equation model indicated that anxiety and depression were highly correlated (*r* = 0.88). Internship experience showed negative associations with emotional exhaustion (β = −0.43), cynicism (β = −0.48), and reduced professional efficacy (β = −0.36). Emotional exhaustion was positively associated with depression (β = 0.23) and anxiety (β = 0.19), while cynicism was linked to both depression (β = 0.06) and anxiety (β = 0.11). Reduced professional efficacy was associated with depression (β = 0.29) and anxiety (β = 0.10) (Figure 3).

## Discussion

This study elucidates a structured pathway linking internship experience to psychological symptoms through burnout among healthcare trainees. Our results demonstrated that younger students reported more favorable internship experiences but also exhibited higher levels of anxiety and depression, highlighting a paradox of enthusiasm coupled with vulnerability. Educational attainment was associated with burnout, with bachelor’s students experiencing higher levels of emotional exhaustion and cynicism than their junior college and vocational counterparts. Importantly, structural equation modeling revealed that burnout fully mediated the relationship between internship experience and psychological symptoms, underscoring its pivotal role as a psychological mechanism in this population.

The association between internship experience and psychological distress observed in this study is consistent with prior research identifying the clinical training phase as a period of heightened psychological vulnerability (10, 12). Previous studies have largely focused on isolated stressors such as workload, patient interactions, or academic demands (11). By applying a comprehensive measure of internship experience, our findings extend this literature by capturing students’ subjective perceptions, thereby providing a more integrative understanding of how educational environments shape mental health outcomes.

Burnout emerged as a central mediating mechanism linking internship experience to psychological symptoms, functioning not only as a consequence of stress exposure but also as a process that amplifies the impact of adverse training environments on mental health outcomes (17, 27–29). While most previous studies relied on correlation or regression analyses, the structural modeling approach enabled identification of both direct and indirect pathways, offering stronger empirical support for the theoretical model that internship experience influences psychological health primarily through its impact on burnout. This pathway underscores the importance of addressing burnout not only as an outcome but also as a mediator that amplifies the effects of adverse educational experiences.

The high comorbidity between anxiety and depression (r ≈ 0.90) observed in this study parallels findings from prior investigations in medical education (17, 30, 31). Such comorbidity suggests that interventions should move beyond targeting isolated symptoms and instead adopt integrated strategies to enhance overall psychological resilience. Together, these results provide converging evidence that the quality of internship experience and the management of burnout are central determinants of healthcare trainees’ mental health.

The findings of this study have several important implications for medical education and student support. Interventions that focus on improving the quality of internship environments, for example by strengthening supervisory guidance, ensuring a balanced workload, and promoting constructive doctor–patient relationships, may help reduce burnout and subsequently alleviate psychological distress among students. Recent evidence from multi-institutional and longitudinal studies indicates that structural factors such as curriculum design, workload distribution, and institutional support play a major role in shaping burnout and psychological outcomes among medical students (32, 33). These findings further support the importance of optimizing training environments to mitigate burnout and improve mental well-being. From an academic perspective, this study contributes in two unique ways. First, it represents the first attempt to apply structural equation modeling to examine the pathways linking internship experience, burnout, and psychological symptoms in Chinese healthcare trainees, thereby confirming the mediating role of burnout with greater methodological rigor than has been previously achieved. Second, the development and application of the self-designed Internship Experience Scale provided a contextually relevant tool to quantify students’ subjective perceptions of internship. This tool fills a gap in the assessment of psychological stress during the clinical training phase in China and offers a new direction for both educational research and practice. Importantly, the present study further demonstrated the robustness and generalizability of the self-developed Internship Experience Scale across different subgroups. The scale was able to distinguish differences in internship experiences across age groups and educational levels, indicating its sensitivity to variations in training contexts and developmental stages. At the same time, the absence of significant gender differences suggests that the scale is not biased by sex and maintains stable measurement properties across male and female trainees. These findings provide additional support for the external validity of the scale and suggest that it can be reliably applied in heterogeneous healthcare trainee populations.

Despite these influences, several limitations need to be noted. Firstly, the cross-sectional design limits the ability to make causal inferences; longitudinal studies or intervention studies are needed to verify the proposed pathological mechanisms. Secondly, the use of convenience sampling from a single teaching hospital may limit the generalizability of the research results. Thirdly, self-administered questionnaires may introduce response bias or social expectation bias. Fourthly, the gender distribution in the sample is unbalanced - approximately 70% of the participants are female. Although no significant gender differences were found in internship experiences, career burnout, or psychological symptoms, the relatively low proportion of male participants may limit the statistical ability to detect gender differences. This gender distribution imbalance may also affect the applicability of the Self-Development Internship Experience Scale in different gender groups. Fifthly, although this study included a diverse sample of medical interns from different backgrounds to reflect the real situation of clinical training environments, this diversity also means that the transitional stress reflected by the indicators we adopted may have different experiences in different groups. Due to the use of convenience sampling and voluntary participation, there may be selection bias. Therefore, the research results may not be fully generalized to all Chinese medical training students or institutions with different organizational structures. Finally, although the self-developed Internship Experience Scale shows good reliability and validity, it still needs to be further verified in larger samples and more diverse gender compositions. Future research should be conducted in larger sample sizes and more balanced gender compositions, focusing on exploring the measurement invariance of this internship scale in different populations and environments and verifying whether the paths identified in this study are affected by the training stage or professional background.

## Conclusion

This study advances the understanding of how educational environments influence the psychological well-being of healthcare trainees. By applying a novel analytical framework and introducing context-specific assessment tools, it provides new insights into the mechanisms linking internship experiences to mental health. These findings highlight the importance of targeted strategies in medical education to foster supportive training environments and promote student resilience.

## Data Availability

The original contributions presented in this study are included in the article/Supplementary material, further inquiries can be directed to the corresponding author.
