# Supplementary material for: Internship experience and psychological symptoms among Chinese healthcare trainees: evidence from structural equation modeling
Source: Front Med (Lausanne). 2026 Jul 14;13:1760463. doi: 10.3389/fmed.2026.1760463 (PMC13407521; doi:10.3389/fmed.2026.1760463)
Supplement: Supplementary file 1 [file Table_1.docx]

Supplementary Table S1.

| Characteristic | Overall  N = 241^1^ | 16-18  N = 30^1^ | 19-21  N = 123^1^ | 22-25  N = 88^1^ | p-value^2^ |
| --- | --- | --- | --- | --- | --- |
| Score_Task_Stress | 4.000 (3.000, 4.500) | 4.000 (3.500, 5.000) | 4.000 (3.000, 4.500) | 3.500 (3.000, 4.000) | 0.009 |
| Score_Teaching_Support | 4.400 (4.000, 5.000) | 4.700 (4.400, 5.000) | 4.400 (4.000, 5.000) | 4.200 (3.800, 4.800) | 0.012 |
| ^1^ Median (Q1, Q3)  ^2^ Kruskal-Wallis rank sum test | | | | | |

Supplementary Table S2.

| Characteristic | Overall  N = 241^1^ | Higher Vocational  N = 31^1^ | Junior College  N = 93^1^ | Bachelor’s Degree  N = 117^1^ | p-value^2^ |
| --- | --- | --- | --- | --- | --- |
| Score_Task_Stress | 4.000 (3.000, 4.500) | 4.000 (3.500, 5.000) | 4.000 (3.000, 4.500) | 3.500 (3.000, 4.000) | 0.004 |
| Score_Teaching_Support | 4.400 (4.000, 5.000) | 4.600 (4.200, 5.000) | 4.400 (4.000, 5.000) | 4.000 (4.000, 4.800) | 0.013 |
| ^1^ Median (Q1, Q3)  ^2^ Kruskal-Wallis rank sum test | | | | | |

Supplementary Table S3.

| Characteristic | Overall  N = 241^1^ | male  N = 72^1^ | female  N = 169^1^ | p-value^2^ |
| --- | --- | --- | --- | --- |
| Score_Task_Stress | 4.000 (3.000, 4.500) | 3.500 (3.000,  4.500) | 4.000 (3.000,  4.000) | 0.14 |
| Score_Teaching_Support | 4.400 (4.000, 5.000) | 4.300 (4.000,  5.000) | 4.400 (4.000, 4.800) | 0.09 |
| ^1^ Median (Q1, Q3)  ^2^ Kruskal-Wallis rank sum test | | | | |

Supplementary Table S4. Factor loadings of the Internship Experience Scale (EFA and CFA).

| Item | Factor | EFA loading | CFA loading |
| --- | --- | --- | --- |
| Q1 | Task-related stress | 0.792 | 0.862 |
| Q2 | Task-related stress | 1.007 | 0.949 |
| Q3 | Teaching support | 0.800 | 0.818 |
| Q4 | Teaching support | 0.725 | 0.774 |
| Q5 | Teaching support | 0.705 | 0.781 |
| Q6 | Teaching support | 0.954 | 0.935 |
| Q7 | Teaching support | 0.952 | 0.914 |

Notes: Values represent standardized factor loadings from exploratory factor analysis (EFA) and confirmatory factor analysis (CFA).
